# Changing ecosystems promote generalism and enhanced heterogeneity in diet composition in the endangered St. Lawrence Estuary beluga

**DOI:** 10.1038/s41598-025-91083-z

**Published:** 2025-02-20

**Authors:** Jory Cabrol, Véronique Lesage, Ève Rioux

**Affiliations:** Maurice Lamontagne Institute, Fisheries and Oceans Canada, Mont-Joli, QC Canada

**Keywords:** Individual diet specialization, St. Lawrence Estuary beluga (*Delphinapterus leucas*), Diet estimation, MixSIAR, Stable isotopes, Mixing models, Trophic interactions, Marine biology, Conservation biology, Stable isotope analysis, Marine mammals, Behavioural ecology

## Abstract

Changes in trophodynamics may affect trophic niche both at the individual and population levels. Using stable isotope ratios, we showed how contrasting oceanographic and trophic conditions in 1997–2003 and 2015–2020 have altered the diet and degree of individual specialization of St. Lawrence Estuary beluga (*Delphinapterus leucas*). The trophic niche of all sex and age classes changed over time, with beluga consuming more small pelagic prey during the first than the second period. Adult male diets differed from that of adult females and juveniles during the first period due to the other prey that were consumed. In 2015–2020, diet contributions by small pelagic prey decreased in all segments of the population and led to marginally significant differences in diet between adult males and females. These dietary changes were concomitant to a diversification of diet at the individual level and to an increase in diet heterogeneity among conspecifics and years within the 2015–2020 period. Whether these patterns emerged from an environment-driven reduction in prey biomass or from an increase in intra- and/or interspecific competition is unknown. Our findings illustrate the importance of considering individuals and not just the population when studying the foraging ecology of endangered species.

## Introduction

With global warming and overexploitation of resources, several marine ecosystems around the world have undergone major changes in their trophic structure and trophodynamics^1,2^. Optimal foraging theory predicts that species continuously adapt their foraging behaviour and prey selection to the spatiotemporal availability of key resources to maximize fitness^3^. However, species or individuals within a species, may vary in their foraging plasticity or capacity to acclimate to changing prey fields. The amount of exploitable alternative resources (i.e., prey diversity), the degree of competition imposed by competitors and factors limiting intraspecific competition, all interplay in defining adaptation success^4–6^. Intraspecific competition is known to have a higher structuring effect on the niche of individuals than the interspecific competition, while the latter tends to limit population niche expansion^4,7^. The degree of intraspecific competition can be modulated through diet specialization or diversification at the individual level^6,8^. In a food-limited environment, individuals may diversify their diet in various ways, for instance by increasing their consumption of resources not fully exploited by conspecifics or by continuing to exploit shared resources while developing marginal feeding behaviors, such as the exploitation of less energetically-optimal prey, atypical resources or newly available resources^9,10^. An example of this is grey seals (*Halichoerus grypus)*which can show cannibalism^11^ or feed on harbour porpoises (*Phocoena phocoena*)^12,13^.

The St. Lawrence Estuary (SLE), in eastern Canada, is a highly dynamic environment with strong salinity and temperature gradients that promote species diversity, differential community structure among sectors and local abundance^14,15^. Like other northern ecosystems, the St. Lawrence has shifted from a relatively cold to a warmer environment with reduced sea-ice cover^16^. These environmental changes combined with overfishing has led to profound modifications of the trophodynamics of the Estuary and Gulf of St. Lawrence over the past decades^16^. Several exploited groundfish stocks have collapsed in the 1990’s; this persistent decline in biomass has not been compensated by increases in other species^17,18^. In addition, populations of several anadromous species specific to the Upper St. Lawrence Estuary (UE), such as rainbow smelt (*Osmerus mordax*) and Atlantic tomcod (*Microgadus tomcod*), have shown evidence of declining abundance^19,20^. In contrast, striped bass (*Morone saxatilis*), redfish (*Sebastes*spp.), and other predators such as several pinniped populations, increased substantially in abundance over the same period^21–23^, and may have altered the top-down control on the communities. These changes have altered the niche breadth and dietary overlap among invertebrates and fish species, but it is unknown how they have affected the trophodynamics and trophic niche of marine mammals^24^.

The beluga (*Delphinapterus*leucas) is part of the SLE community. Despite the implementation of conservation measures to reduce the risk of extinction since the 1980’s^25^, this small isolated population estimated at 1,500 – 2,200 individuals in 2022 has failed to recover^26,27^. While the primary cause for the initial decline is historical overharvest, the reasons for the current lack of recovery remain uncertain, but likely include a combination of climatic and anthropogenic stressors, several of which are directly or indirectly mediated through food quality, quantity, or access^26,27^. However, the paucity of information on SLE beluga diet precludes the identification of key resources and therefore, impairs our ability to determine the capacity of SLE beluga to adapt their feeding strategies to cope with changes in prey availability and potential increase in competition for shared resources.

**Fig. 1:**
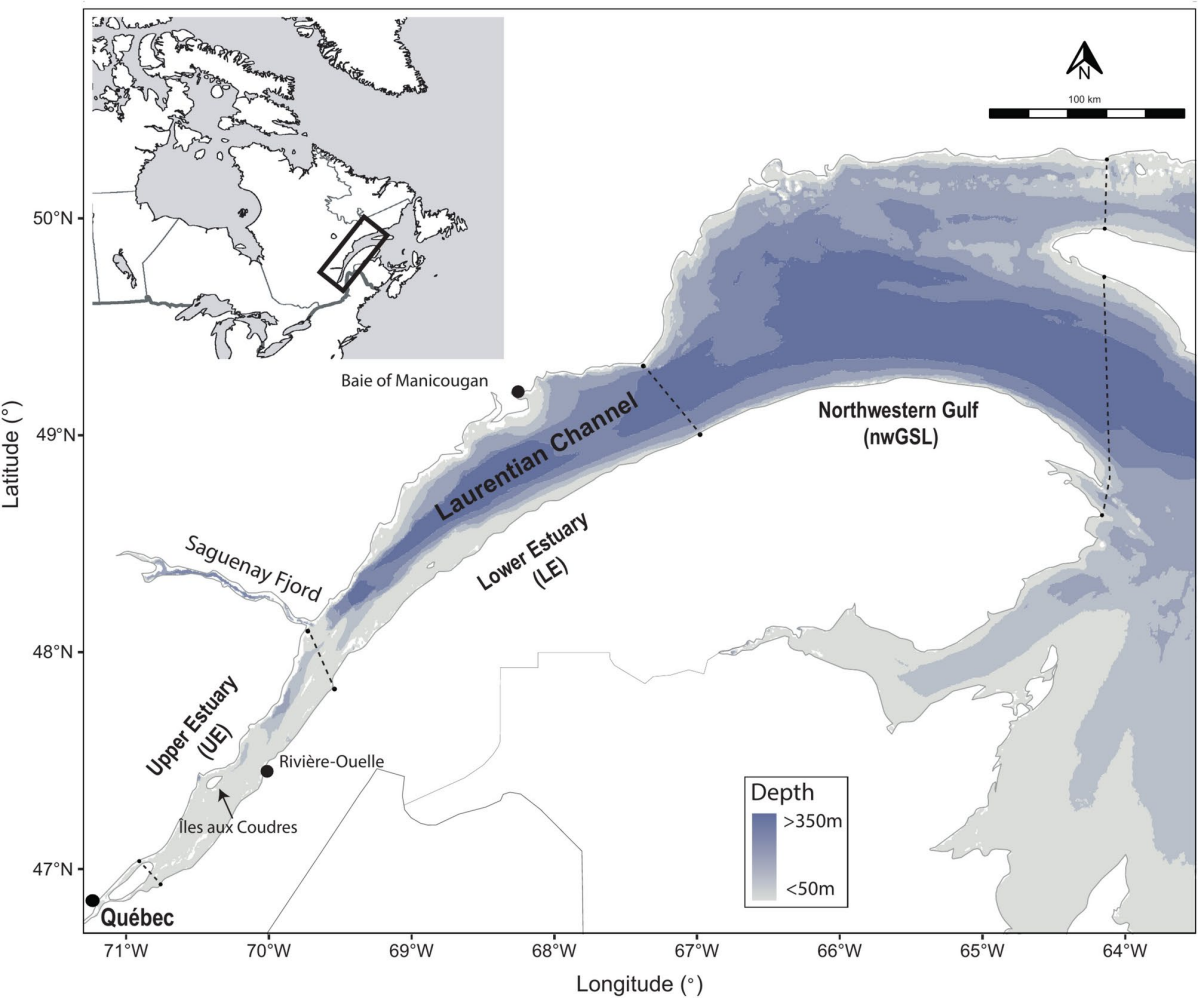
Study area of the endangered St. Lawrence Estuary beluga population. Dashed lines indicate boundaries between the Upper Estuary (UE), the Lower Estuary (LE) and the northwestern Gulf of St. Lawrence (nwGSL). Bathymetric data were provided by the Canadian Hydrographic Service.

The beluga is considered an opportunistic species, and SLE beluga are no exception, consuming a wide variety of prey ranging from benthic and pelagic invertebrates to demersal and pelagic fishes^28,29^. While the summer habitat of SLE beluga is well defined, uncertainties persist on the full extent of their seasonal movements and distribution outside of summer. Data from aerial surveys indicate that they heavily use the SLE and part of the Saguenay Fjord year-round, and occur seasonally in the northwestern Gulf of St. Lawrence^27,30^ (Fig. 1), suggesting that they have access to different prey assemblages along the estuarine gradient. Most of our current understanding on the realized trophic niche of SLE beluga comes from stomach contents collected 80 years ago from sites only occasionally used by beluga^28^, and from digestive tracks collected more recently from beluga found dead largely with empty stomachs^29^. In the SLE, male and female beluga show a partial spatial segregation during summer, with adult females and juveniles using intensively warmer and shallower waters, and adult males occupying some of these sectors but also deeper and colder waters^31,32^. Although, this spatial segregation might limit competition by exposing sex and age classes to different prey assemblages^29,33,34^, it also means that not all segments of the population experience similar changes in trophodynamics over time.

The main objective of this study was to define how the realized trophic niche of SLE beluga has changed over the past decade in the context of major climate and anthropogenic perturbations. A stable isotope approach using carbon (δ^13^C) and nitrogen (δ^15^N) isotope ratios was applied to gain insights into these changes both at the individual and population levels. Dietary plasticity was examined by estimating diet composition using Bayesian mixing models (MixSIAR)^35^, degree of diet specialization (Ɛ)^36^, and heterogeneity in resource selection among individuals. These metrics were compared among two periods of contrasting environmental conditions: i) the 1997 – 2003 period (2000’s), which followed the collapse of the groundfish fishery with a system dominated by invertebrates and pelagic fish; and ii) the 2015 – 2020 period (2020’s), which was characterized by more temperate condition, reduced sea-ice cover, a decrease of some pelagic species, and an increase in potential competitors^16,37,38^.

## Methodology

### Beluga sampling

Carcass beluga sampling were authorized in accordance with relevant guidelines and permits delivered under the Species at Risk Act (2019 onwards) or the Fisheries Act (all years). Overall, 96 well-preserved to moderately decomposed SLE beluga carcasses (freshness codes < 4)^39^ were sampled over the periods 1997 – 2003 (*n* = 52) and 2015 – 2020 (*n*= 44) following a standardized protocol that was consistently applied over time^40^. The annual and seasonal distribution of sampling effort was comparable between the two periods (see Fig. S1 and Table S1). Muscle tissue was collected from the sun-protected flank of the animal and kept frozen until analyses. Sex was determined visually, while age was determined by counting growth layer groups (GLGs) on longitudinal tooth sections, assuming deposition of one GLG per year^41^. Only individuals aged two years or older were included in the study to reduce bias associated with lactation on stable isotope values^42^. Individuals aged 8 + GLGs were considered to be adults^43^.

### Prey sampling

Potential prey were selected using multiple sources of information: 1) stomach content analyses from SLE beluga hunted in the 1930’s^28^; 2) digestive tracts of individuals found dead since the 1980’s^29^; 3) local prey abundance^21,37^; and 4) field observations of feeding behavior (Groupe de Recherche et d’Éducation sur les Mammifères Marins, Tadoussac, QC, Canada, unpublished data). A total of 926 specimens from 17 taxa (see Table S2 and S3 for details) were sampled from various regions of the SLE beluga range, including the UE, the Lower Estuary (LE) and the northwestern Gulf of St. Lawrence (nwGSL; Fig. 1). Sampling took place during the ice-free season (April to November) in both 1995 – 2003 (*n* = 248) and 2015 – 2020 (*n*= 678). Prey were obtained from regular monitoring and other research programs of Fisheries and Oceans Canada, from local fishermen, and collaborators from universities and other governmental organizations. Sampling methods, including sampling locations, specimen identification, conservation and sub-sampling were consistent over time. Specimens were captured using various techniques ranging from fixed weirs to pelagic and benthic trawls^24^. Fish specimens were within the size range of those found in SLE beluga digestive tracts^29^, with average lengths of 18.6 ± 7.6 cm in the 2000’s and 20.7 ± 7.4 cm in the 2020’s. Muscle tissue was excised dorso-laterally from each fish specimen and from mantle for squids.

### Stable isotope analyses

Tissues were freeze-dried for 48 h and grounded to a powder before isotope analyses. Lipids have a lower δ^13^C value compared to protein, which introduces undesirable variability in lipid-rich species and affects diet and niche width estimations^44^. Lipids were thus extracted from beluga and prey samples for δ^13^C determination using the Folch method with a chloroform/methanol solvent mix (2:1, v/v)^45^. In the 1990’s, the effect of lipid-extraction on δ^15^N values was not documented^46^, and thus, δ^15^N were determined from lipid-extracted samples. To account for this bias, bulk δ^15^N values were restored for these samples using mathematical equations specifically developed for SLE beluga and their prey^47^. For the 2020’s period, δ^13^C and δ^15^N values were determined from separate aliquots, i.e., lipid-extracted for δ^13^C and untreated for δ^15^N.

Stable isotope analyses were performed using a continuous-flow stable-isotope mass spectrometer coupled to a Carlo Erba elemental analyzer (CHNS-O EA1108). Carbonates from Vienna Pee Dee Belemnite limestone and atmospheric nitrogen were used as standards for δ^13^C and δ^15^N, respectively. Stable isotopes are expressed in δ notation as the deviation from international standards in parts per thousand (‰) according to the following equation:

δX = [(Rsample/Rstandard) – 1] × 1000(1).

where δX is δ^13^C or δ^15^N, and R is the corresponding ^13^C/^12^C or ^15^N/^14^N ratio. Analytical errors based on replicate analyses of samples (*n* = 462) were similar between periods (median: 0.13‰ for δ^13^C; 0.17‰ for δ^15^N).

### Data analyses

The Suess effect is a phenomenon known to reduce δ^13^C values through the release of large quantities of CO_2_into the atmosphere when burning fossil fuels^48^. Due to the carbon cycle, this depletion propagates through the food web and so, must to be accounted for when analyzing time series^49^. The SuessR package^50^ was used to correct for the Suess effect, as well as the Laws effect, which accounts for the variable CO_2_ exchange rate with water temperature on aqueous CO_2_concentrations^51^. To limit the volume of mathematical corrections, the Suess effect correction was applied by centering beluga and prey samples within a given period, on the year with the highest sample size, i.e., 2002 for 1995 –2003 and 2020 for 2015 –2020 and using preset value for the North Atlantic region from SuessR package^50^.

As the first step in data analysis, sampling period (2000’s vs 2020’s), location (UE, LE, nwGSL), and sampling year were examined for potential effects on prey isotopic signatures using permutational multivariate analyses of variance (PERMANOVA) and pairwise comparisons, using the R package ‘Vegan’ (version 2.6–8)^52^. Significance for all pairwise tests was evaluated using the Benjamini & Yekutieli adjustment method to limit type I error^53^. The PERMANOVA was performed using Manhattan distance, 9999 permutations and partial sum of squares. The model was validated for bivariate normality, an underlying assumption of mixing models^35^. Prey that were deemed similar isotopically and ecologically were combined a priori for each period to avoid a lack of convergence, and overcome the problem of poorly specified mixing systems often associated with a high number of potential food sources^35^. The absence of outliers was confirmed in the final prey selection using a dispersion approach^54^. All analyses were performed using the R programming language (R core team; version 4.3.1)^55^. Data are presented as mean ± standard deviation. All data are available as supplementary material.

### Diet estimation

#### Model selection

The relative contribution of the various potential prey or prey groups to beluga diet was estimated using the R package “MixSIAR” (version 3.1.12) a Bayesian stable isotope mixing model^56^. To account for the change in niche breaths and isotopic signatures that was documented over time in some beluga potential prey^24^, diet composition was estimated separately for each time period using two distinct sets of prey and separate model runs. Instead of selecting a single model believed to be the “most biologically relevant”, a model selection approach was applied, where models with no covariates (Null model), with fixed effects only (sex/age classes: juveniles, adult males and females; sampling year: 5 or 6 levels; individual beluga: *n* = 96), with random effects only or all biologically plausible combination were fitted to the data to assess their influence on diet estimates. Eight candidate models were formulated for each period (Table 1). To evaluate their relative support, the widely applicable information criterion (WAIC) and approximate leave-one-out cross-validation (LOO) were used since all models in each period were fitted to the same data. WAIC and LOO were preferred over the deviance information criterion (DIC). Both metrics are more robust and better suited for Bayesian mixing models^35^, they are insensitive to model parametrization, applicable to singular models and do not produce negative estimates of the effective number of parameters^35,57^.

**Table 1:** Ranking of candidate models used to estimate diet of SLE beluga. Widely applicable information criterion (WAIC) and approximate leave-one-out cross-validation (LOO) information criteria are presented as mean ± standard deviation. The ∆LOOic / ∆WAIC = compare the LOOic or WAIC value to the most parsimonious model for each time period (in bold). ^a^ = Random effect.

Explanatory variables	Period	LOOic	∆LOOic	WAIC	∆WAIC	Error term
Null model	2000’s	56.9 ± 22.4	13.0	56.9 ± 22.5	15.3	Residual x Process
	2020’s	145.4 ± 15.1	16.1	145.3 ± 15.0	22.3	
"Sex and age classes"	2000’s	45.2 ± 19.5	1.3	44.8 ± 19.3	3.2	Residual x Process
	2020’s	148.7 ± 16.6	19.1	148.5 ± 16.5	25.5	
Individual^a^	2000’s	66.0 ± 9.5	22.1	61.8 ± 9.1	20.2	Process only
	2020’s	132.4 ± 19.3	2.8	120.6 ± 18.0	2.4	
Year	2000’s	63.9 ± 21.0	20.0	63.0 ± 20.7	24.5	Residual x Process
	2020’s	133.4 ± 18.7	3.8	133.0 ± 18.6	10.0	
"Sex and age classes"(individual^a^)	2000’s	**43.9 ± 19.6**	0	**41.6 ± 18.7**	0	Residual x Process
	2020’s	142.8 ± 16.2	13.2	137.7 ± 15.7	14.7	
Year (individual^a^)	2000’s	60.0 ± 20.4	16.1	56.2 ± 19.0	14.6	Residual x Process
	2020’s	**129.6 ± 19.7**	0	**123.0 ± 18.5**	0	
Year x "Sex and age classes"	2000’s	54.6 ± 19.6	10.7	52.4 ± 18.7	13.9	Residual x Process
	2020’s	140.0 ± 19.2	10.4	138.7 ± 18.9	15.7	
Year x "Sex and age classes" (individual^a^)	2000’s	74.1 ± 19.5	30.2	49.7 ± 18.2	8.1	Residual x Process
	2020’s	136.7 ± 18.0	7.1	129.0 ± 17.1	6.0	

#### Model parametrization

Model parameterization employed a non-informative Dirichlet prior distribution and prey carbon to nitrogen elemental ratios (C:N) were used to inform concentration dependencies^58^. All models utilized the multiplicative error terms as defined in Stock et al.^35^, except for the model that included only the individual as a covariate, and for which residual error-only was selected^56^. The goodness-of-fit of the mixing model for each period was validated using simulated mixing polygons approach before diet estimation^59^. Convergence was considered achieved when all R-hat values were less than 1.05 (Gelman-Rubin convergence diagnostic). Additionally, to determine the effect of the prior selection on diet estimation, prior and posterior distributions of diet estimates were plotted and visually compared for both periods (Fig. S2). A PERMANOVA approach was employed to identify estimated diet that were statistically different among the defined sex and age classes and the two study periods.

Currently, there are no trophic discrimination factors specific to beluga muscle. Therefore, discrimination factors (∆^13^C = 1.3 ± 0.5 and ∆^15^N = 2.4 ± 0.5) for muscle obtained from captive seals fed herring were used as a surrogate^60^. These values are comparable to the discrimination factors obtained from the plasma of killer whales (*Orcinus orca*) and bottlenose dolphins (*Tursiops truncatus)*^61^, and were also in the range of values found for mammals as recently reviewed in Stephens et al.^62,63^. The trophic discrimination factors used for nitrogen is also consistent with the range of values (i.e., 2 – 3.1‰) predicted using the mean isotopic value of hypothetical prey of SLE beluga^64^.

### Individual diet specialization

The dietary specialization index (Ɛ)^36^reflects the degree of prey selectively of individuals within a population. Ɛ was calculated by comparing the diet of each individual relative to a hypothetically perfect generalism (i.e., equal selection of all prey resources). Diet for each individual were obtained from compositional diet estimates obtained from isotopic mixing models^65^. Posterior contributions from pelagic prey from different areas were combined to calculate Ɛ. Calculations were performed in an Euclidean space, where 0 corresponded to ultra-generalism and 1 corresponded to an ultra-specialism. .

### Inter-individual variability in prey selection

Heterogeneity in prey selection among individuals of a specific sex or age class was assessed separately for each period using the multivariate PERMDISP procedure^52^. Briefly, compositional diet estimates were used to compute the average distance between an individual and the centroid of its group (e.g., females in the 2000’s) in an Euclidean space. Statistical differences among groups and periods were assessed using a PERMANOVA, with results illustrated using multidimensional scaling^66^.

## Results

### Isotopic signatures of prey

**Fig. 2:**
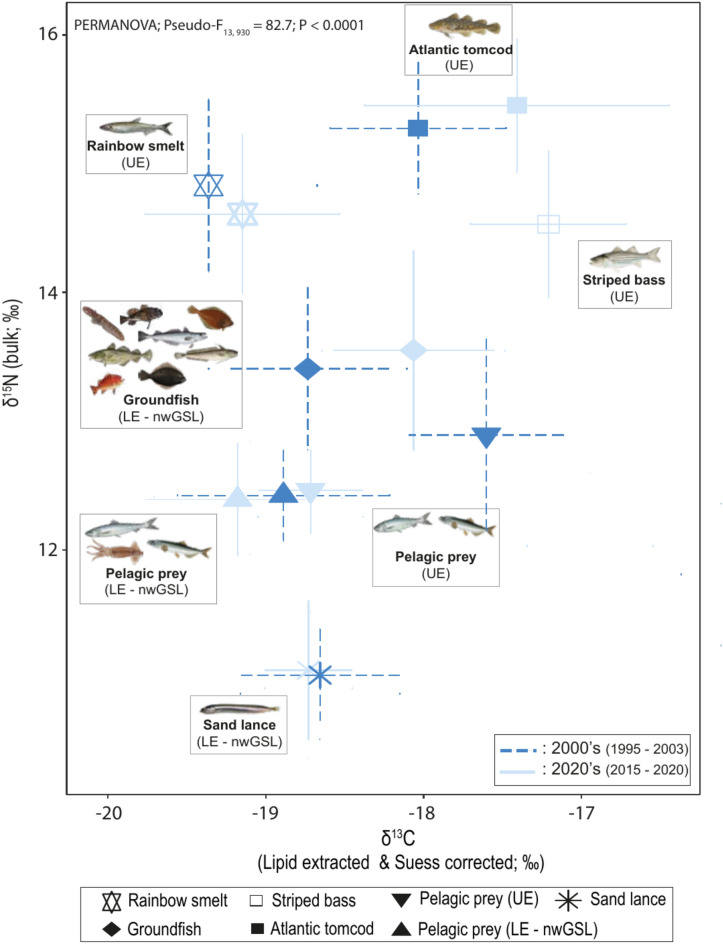
Isotopic signatures of prey groups (mean ± SD) used in mixing models for the periods 2000’s (light blue solid line) and 2020’s (dark blue dashed line). See Table S2 in supplementary material for details on prey group taxonomical composition and sample sizes, and Table S7 for isotopic values. All images are from open sources or were provided by C. Nozères (Fisheries and Oceans Canada, Mont-Joli, QC).

The seventeen potential prey taxa sampled during the two study periods were aggregated into seven functional groups (Fig. 2), which all differed statistically for a given period based on their bivariate isotopic signatures. Detailed statistics on prey differentiation are presented in Tables S4 to S6. The seven groups were: 1) groundfish, a group consisting in 6 to 9 taxa (see Table S2 for taxonomical details) with similar ecological role and comparable isotopic signatures; 2) Atlantic tomcod from the UE; 3) striped bass also from the UE; 4) pelagic prey from the LE and nwGSL, which comprised capelin, Atlantic herring (*Clupea harengus*) and northern shortfin squid (*Illex illecebrosus*), three species that were isotopically indistinguishable (PAIRWISE, all P > 0.79); 5) pelagic fishes from the UE, which included Atlantic herring and capelin, two species also isotopically indistinguishable (PAIRWISE, all P > 0.46) during both time periods; 6) sand lance (*Ammodytes* spp.) from both the LE and nwGSL; and 7) rainbow smelt (*Osmerus mordax*) from the UE (Fig. 2; Table S2 and S3).

Isotopic signatures varied between periods for some of the functional groups. While they remained relatively unchanged for sand lance, rainbow smelt and pelagic prey from the LE/nwGSL (Table S6), a low to moderate ^13^C enrichment with little change in δ^15^N values was noted for Atlantic tomcod and groundfish. A more pronounced decrease in both isotope ratios was noted in the 2020’s for pelagic prey from the UE, leading their isotopic signatures to become less differentiated from pelagic prey from the LE (Fig. 2), although their bivariate isotopic signatures still differed significantly (P < 0.0001; Table S5).

### Best models for diet estimation

The LOO and WAIC metrics were consistent in their assessment of the best supported model for each period. Best models included individuals as a random effect in both time periods, indicating intraspecific variation in diet composition. While the best model for the 2000’s included "sex and age classes" as a covariate, the model best fitting the data in the 2020’s included a different covariate, i.e., “sampling year” (Table 1). Competing models (LOO and WAIC ≤ 4) included only “sex and age classes” in the 2000’s and only the random effect in 2020’s, suggesting that the random effect might have been less important in the 2000’s than the 2020’s period (Table 1). No high negative or positive correlations (r^*2*^ > 0.80) were observed between prey groups in either time period, indicating that models were successful in differentiating contributions from all potential prey groups (Fig. S3).

### Diet estimations

**Fig. 3:**
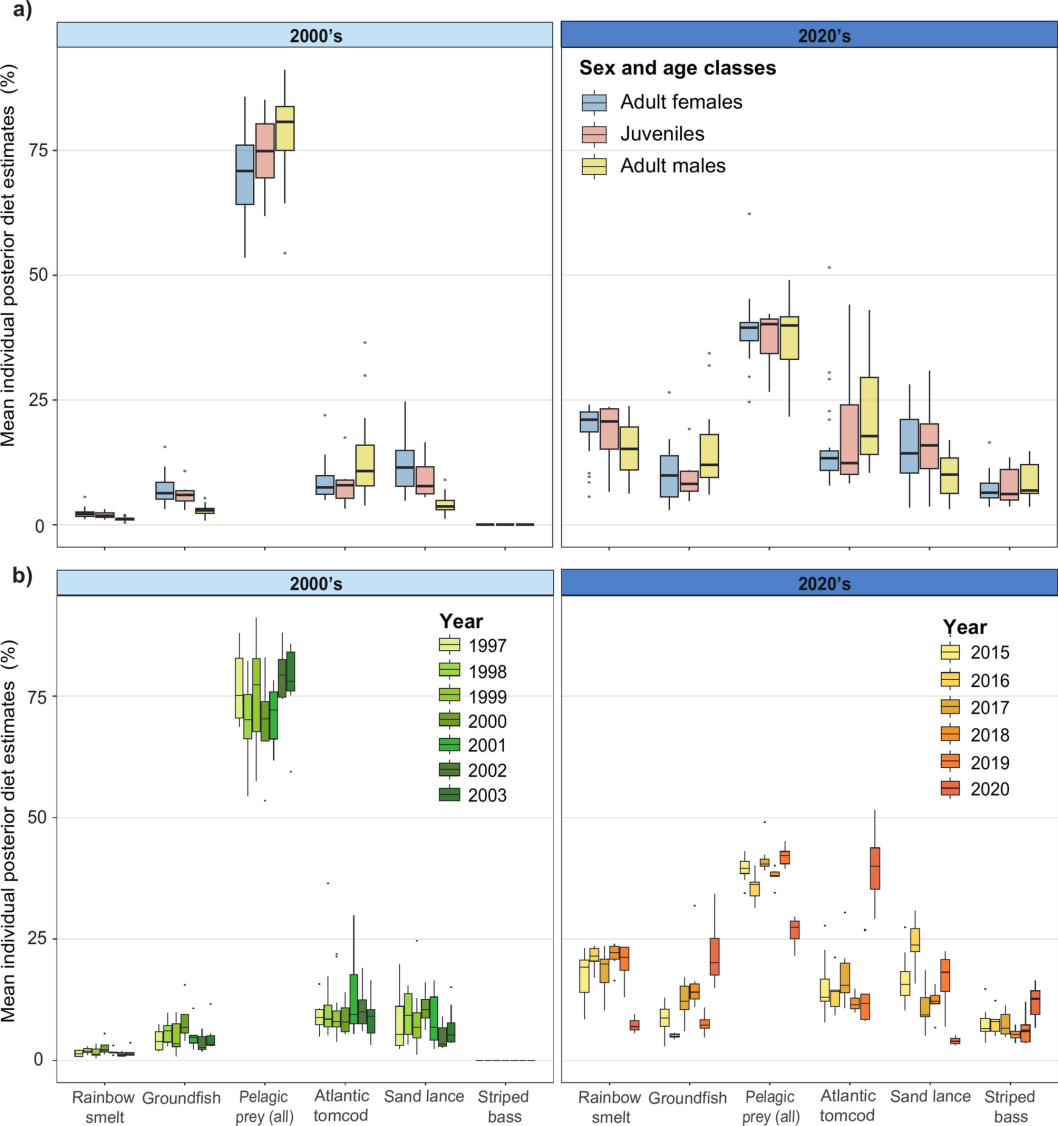
Distribution of the mean individual diet proportions for each potential prey or prey group as estimated by isotopic mixing models for **a)** each sex and age classes and **b)** each year during the 2000’s (light blue) and 2020’s (dark blue). Pelagic prey (all) represented the summed contributions from all locations (UE, LE and nwGSL). Mean ± SD values are detailed in Tables S8 and S10 in the supplement.

Pelagic prey dominated the diet of all sex and age classes during both periods, and was consistently higher in the 2000’s (67 – 82% depending on sex and age classes) than the 2020’s (33 – 42%; Fig. 3). Other prey groups increased in importance in the second period, leading to a change in diet composition over time (Fig. 3a).

**Fig. 4:**
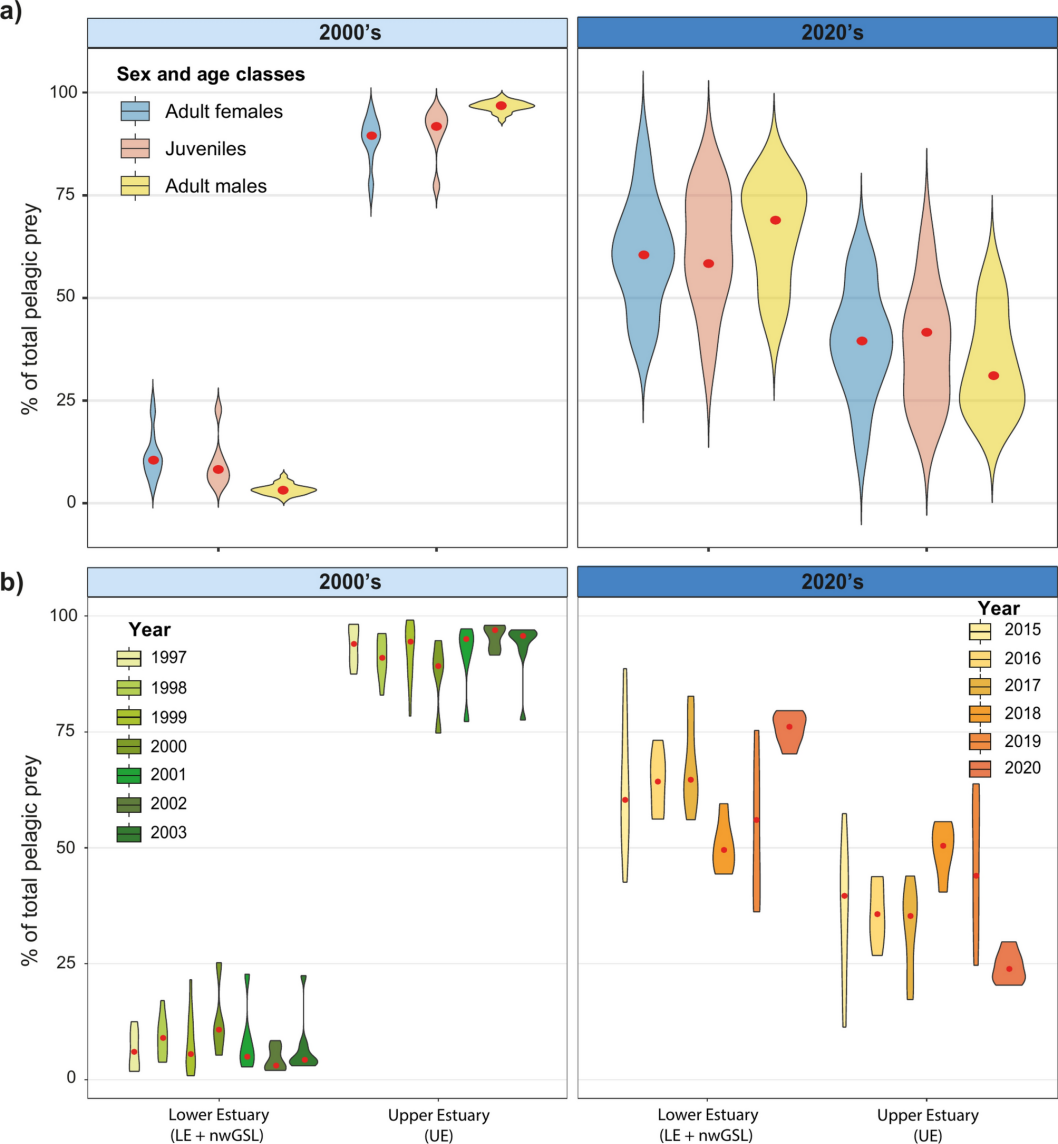
Proportion of the pelagic prey (in %) in SLE beluga diet coming from the Upper Estuary (UE) and the Lower Estuary (LE) / northwestern Gulf of St. Lawrence (nwGSL), presented for a) each sex and age classes, and b) each year during the 2000’s (light blue) and 2020’s (dark blue).

In the 2000’s, adult females and juveniles had a similar diet (PAIRWISE, P = 0.35), which differed from that of adult males (PAIRWISE, both P ≤ 0.001; Fig. 3). During that period, most (88.4 – 96.6% on average) of the pelagic prey consumed by the different sex and age classes originated from the UE (Fig. 4). In addition to pelagic prey, adult females and juveniles also consumed sand lance, and to a lesser extent groundfish and Atlantic tomcod, whereas adult males consumed Atlantic tomcod (13% on average; Fig. 3 and Table S8 and S9), and to a lesser extent sand lance and groundfish.

In the 2020’s, diet of juveniles continued to be similar to that of adult females (Fig. 3). However, diet differences that were observed with adult males in the 2000’s attenuated in the most recent period to become statistically non-significant (PAIRWISE, P = 0.25; Fig. 3). The contribution of pelagic prey from the LE and nwGSL also surpassed that of the UE for all sex and age classes, accounting on average for 60 – 66% of the total consumed for this prey group (Fig. 4). Females, and especially juveniles, consumed a higher proportion of sand lance and rainbow smelt compared to the 2000’s, with these two prey together representing up to 39% of their total diet. For adult males, the decrease in consumption of pelagic prey was mainly compensated by a 7- and fivefold increase in the consumption of rainbow smelt and groundfish, respectively. The striped bass which was virtually absent from the ecosystem in the 2000’s, and thus not sampled, was consumed by all sex and age classes in the 2020’s, representing now between 5 to 11% of the total diet.

One striking difference between the 2000’s and the 2020’s was the much larger interannual variability observed in diet composition during the latter period (Fig. 3b). In contrast to the 2000’s, interannual variability in the 2020’s surpassed that observed among age and sex classes. This was particularly true during the last year of study (2020), when Atlantic tomcod and striped bass increased, and pelagic prey (all sectors confounded) decreased in importance in the diet of all three sex or age classes compared to previous years in this period, and when sand lance and rainbow smelt became nearly absent from the diet.

### Degree of individual specialization

**Fig. 5:**
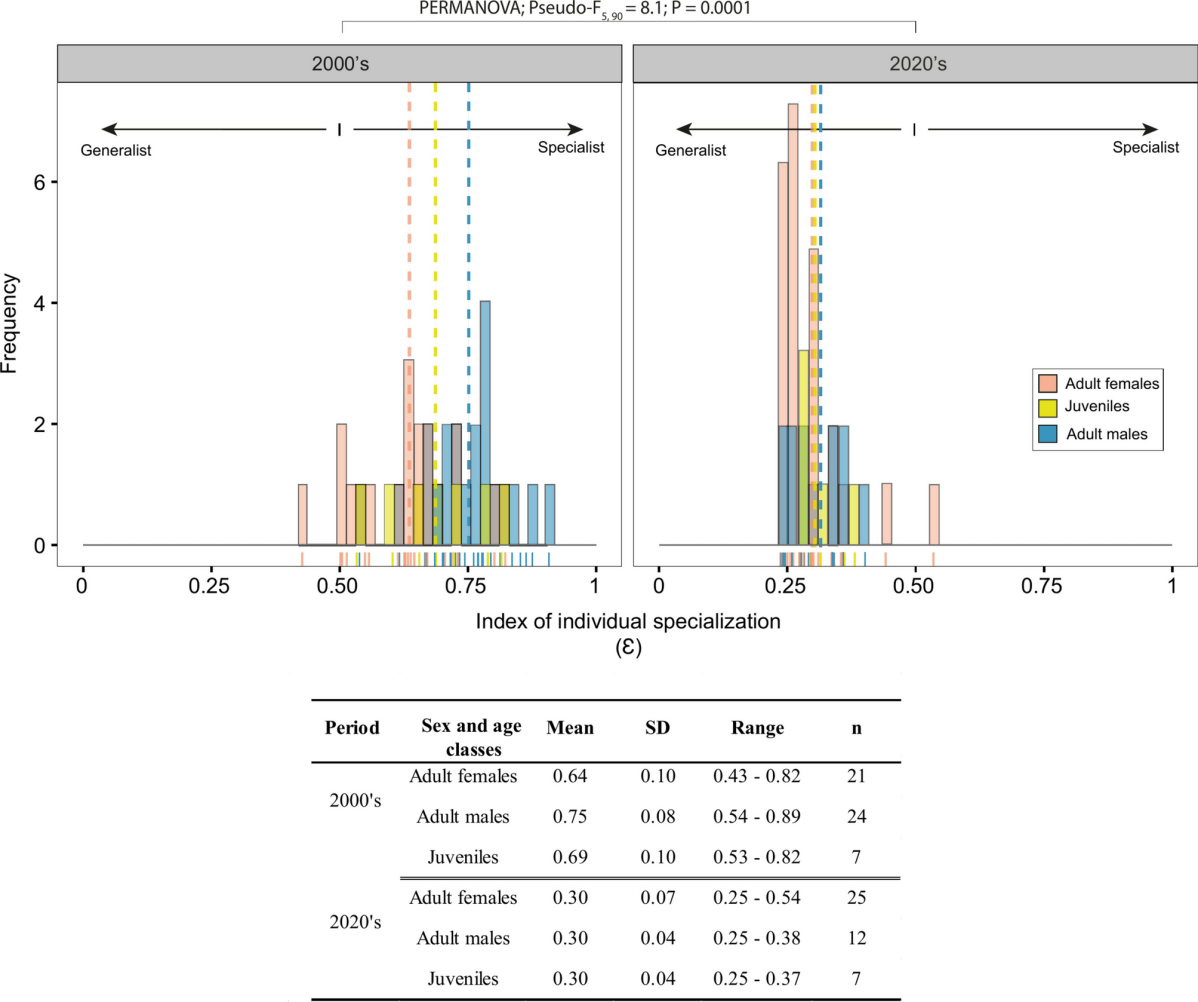
Frequency distribution of the specialization index (Ɛ) for adult females (pink), adult males (blue) and juveniles (yellow) for the 2000’s and 2020’s based on estimates from isotopic mixing models. A value of 0 corresponded to an ultra-generalist while a value of 1 corresponded to an ultra-specialist.

The observed changes in diet composition between the two periods were concomitant to significant changes in degree of specialization of individual beluga from all sex and age classes (Fig. 5). The specialization index (Ɛ), which was with one exception consistently of 0.50 or more during the 2000’s (range: 0.43 – 0.89), decreased considerably during the 2020’s, both in values and in range (range: 0.25 – 0.54; Fig. 5) compared to the 2000’s. The decrease was particularly pronounced for adult males, for whom Ɛ declined from an average of 0.75 ± 0.08 in the 2000’s to 0.30 ± 0.04 in the 2020’s. In comparison, Ɛ decreased from 0.64 ± 0.10 in adult females, and from 0.69 ± 0.09 in juveniles, to a value of 0.30 (SD of 0.07 and 0.04, respectively) for both classes during the 2020’s. Statistically, all three sex and age classes became indistinguishable on the basis of their Ɛ during the most recent period (all P > 0.87). This was not the case in the 2000’s, when adult males had significantly higher Ɛ than adult females (PAIRWISE, P = 0.015); juveniles had intermediate and overlapping Ɛ with adult males and females (PAIRWISE, all P > 0.55; Fig. 4).

### Diet heterogeneity among individuals

**Fig. 6:**
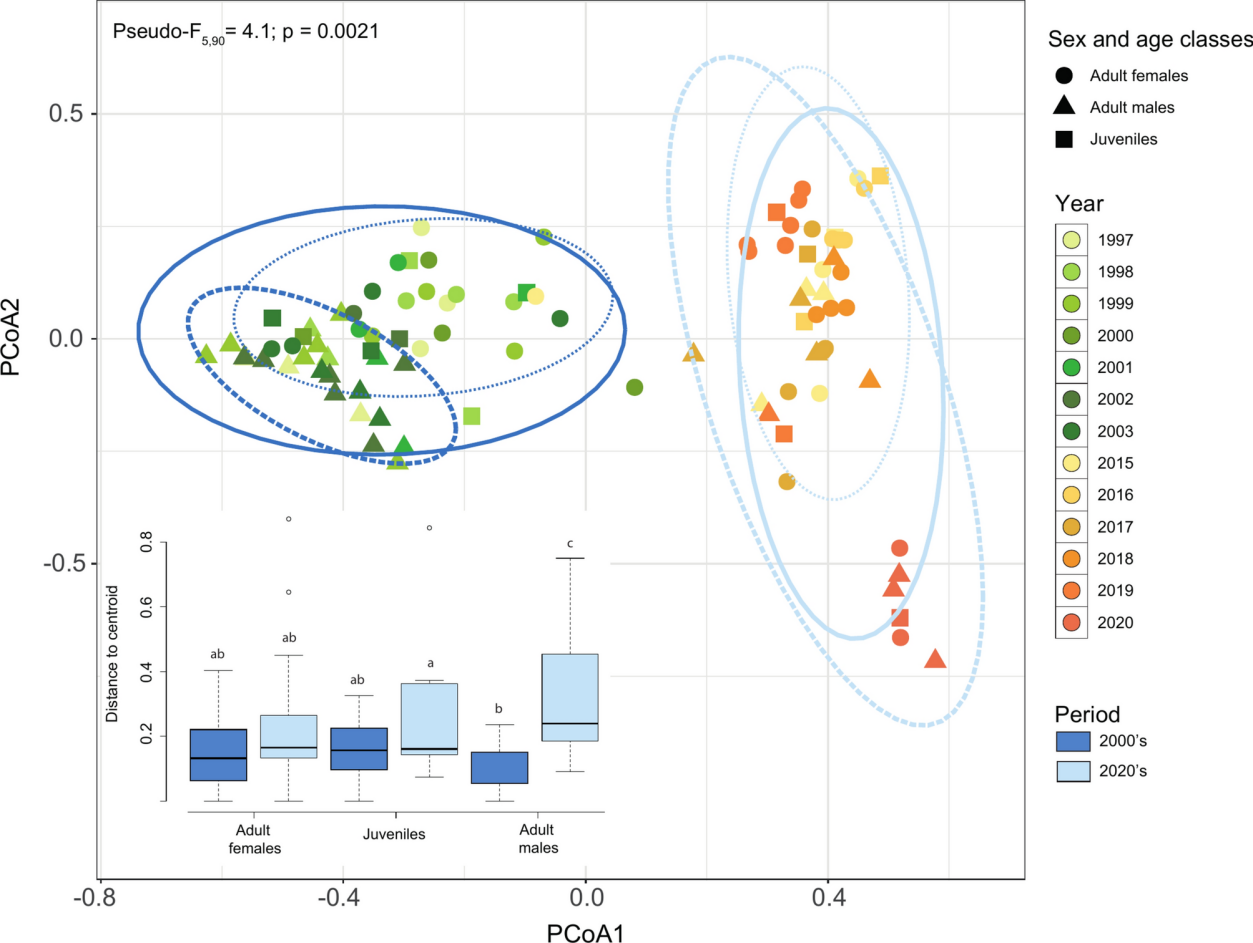
Interindividual heterogeneity in diet composition as expressed by the average distance of each individual to the centroid of their sex and age class, i.e., adult females (circles), adult males (triangle) and juveniles (square) for both the 2000’s (light blue) and 2020’s (dark blue) and for each year within each period. Different letters indicate significant differences based on PAIRWISE comparison. Detailed interactive 3D scatterplot is also available in the Supplementary Fig. S4.

The degree of inter-individual variability in diet composition, estimated as the dispersion of individuals around the centroid of their respective sex or age class, was similar in adult males, adult females and juveniles in the 2000’s. Diet heterogeneity was of the same magnitude in adult females and juveniles during the 2020’s, but was larger in adult males (Fig. 6). In the 2000’s, the observed diet heterogeneity was unrelated to a year effect, whereas in the 2020’s, both individuals and yearly variations in diet contributed to the observed pattern (Fig. 6).

## Discussion

Ecosystems around the world have been subject to profound changes over the past few decades, but long-term data are often lacking to understand how species respond to these changes. Here, long-term sampling of both a consumer and its potential prey has shown how SLE beluga adjusted their foraging strategies at both the individual and population levels in response to changes in the trophic structure of their environment. Contrasts in diet composition and degree of specialization between periods revealed that SLE beluga changed their diet in several ways. They switched from a more specialized diet containing mostly pelagic prey, which were thought to be more abundant in the 2000’s, to a more diversified diet with higher heterogeneity among conspecifics. Combined with the decrease in individual diet specialization in the 2020’s, this suggests that beluga became less selective in their prey consumption in recent years when ecosystem structure and prey abundance are suspected to have changed considerably, a phenomenon also observed in fin whales in this region^65^.

SLE beluga occupy a variety of habitats in the SLE and nwGSL during their annual cycle^27^**.**These habitats differ isotopically and in community structure even within the SLE^24,33^. Areas of high use or of high residency have been identified within the SLE beluga annual range^30,67^**.**While these appear to be used mostly as foraging areas^31,68^, they vary greatly in terms of their environmental conditions (e.g., salinity, turbidity, temperature), suggesting that they support different prey assemblages and/or abundances. Beluga from different sex and age classes exhibit partial segregation during summer, and some degree of spatial structure and site fidelity within their respective summer ranges^31,69,70^. Adult females and juveniles generally occur in association, with herds of adult males being either intermixed with older juveniles or observed on their own^31,32,68^. While herds of adult females and juveniles share some areas with herds of adults (presumably males), for instance in or near the Saguenay Fjord or the shallower waters along the south shore of the LE^71^, they are rarely observed in the deeper waters of the Laurentian Channel during summer^31^. The spatial structure of the population, combined with the variability in foraging habitat characteristics, may lead to additional complexity when trying to identify key prey species or the habitats likely important to different segments of the population, or the population as a whole. The spatial and taxonomical resolution of the bulk isotopes remains, however, too coarse to pinpoint the specific origin of an ingested prey within the UE, LE or nwGSL and thus, to examine SLE beluga foraging behaviour at a finer scale within their foraging habitats. To test this hypothesis, Sulfur stable isotope analysis and compound specific isotope analysis on amino acids or fatty acids might prove useful due to their higher sensitivity to characteristics of carbon sources^72,73^.

The variability in diet composition observed among individuals of a given sex or age class in our study is consistent with the expectation that individuals have access to a variety of foraging habitats with varying prey abundance and composition. For instance, the deep waters of the Laurentian Channel (Fig. 1), where adult males tend to aggregate, are likely to be suitable habitat for groundfish species and squid^21^**.** Therefore, a greater contribution of these species to the diet of adult males might be expected given their overlapping distributions. This was observed for groundfish in the 2020’s, although not in the 2000’s. Part of the observed diet heterogeneity among individuals of the same class may also have emerged from interannual and seasonal changes in prey availability within these specific habitats. Similarly, fine scale prey abundance and distribution are unknown and thus, limit our capacity to determine whether the observed interannual change in prey consumption, especially during the 2020’s, is related to prey availability or reflects distinct foraging strategies.

Historical data suggested that in the spring and early summer, spawning pelagic fishes such as capelin dominated the diet^28^, with groundfish species such as cod (either *Gadus morhua* or *G. Ogac*, or both), and other species such as sand lance, squid and possibly Atlantic herring possibly becoming more important in the diet later in the summer when water warms up, or during the fall^28^(e.g., rainbow smelt). If groundfish species indeed become important in beluga diet only in mid- to late- summer, and considering that more than half of the beluga were collected before August, then it is possible that the importance of groundfish in summer diet was underestimated to some extent in our sample. Our sample size (96 beluga) remained too small to explore this question or the seasonal variation in diet within each of the two periods. On the other hand, digestive tracts provide information on the last few meals, and tend to underestimate contributions from prey with small or erodible hard parts^74,75^. Biases associated with this method may also have contributed to the observed discrepancies between diet estimates. An illustration of this, is the discrepancy observed between the high values estimated for small pelagic prey in our stable isotope study and that from the 1930’s using stomach contents from freshly killed beluga^28^, and the low values estimated in a more recent study using digestive tracts from beluga recovered a few days after^29^. The stable isotope approach using muscle tissue provides an integrated view of diet over several weeks up to a few months, which can be seen as an average diet over this period^76^. Our sampling scheme, where more than half of the beluga were collected before August (Fig. S1), likely allowed to capture well beluga diet in the spring and early summer. Diet estimations were in agreement with historical hunter reports and findings from the 1930’s^28^, confirming that the diet of all sex and age classes is dominated by pelagic prey, which consisted of two (UE) to three (LE and nwGSL) species in our study (i.e., capelin, Atlantic herring and northern shortfin squid). However, the decline in the consumption of pelagic prey in the 2020’s is puzzling, as it was associated with an increased contribution of pelagic prey from the LE and nwGSL, and also a change in δ^13^C values of pelagic prey from the UE toward values more typical of the LE, suggesting that capelin and herring changed their habitat use overtime. Given that the UE is mostly occupied by adult females accompanied by calves and juveniles during summer^31,71^, such a shift might have important bearing for the population and potential for inter- and intra-specific competition.

Estimating diet using isotopic mixing models is also challenging, especially for generalist species when the number of potential prey are numerous and/or isotopically similar. These conditions trigger a need for a priori prey selection and grouping, limiting our capacity to estimate the contribution of individual prey to the diet. For pelagic prey from the LE, which comprised capelin, Atlantic herring and northern shortfin squid, historical and contemporary diet from digestive tracts suggest that all three species might be preyed upon by SLE beluga, with capelin being more important in spring and early summer, squid increasing in importance during summer, and Atlantic herring being more important during spawning in May, and possibly again later during summer^28,29^. In the SLE, capelin is considered an important forage species for many predators due to its high energetic content^77^, relative abundance^37^, and ubiquity^78^. Direct evidence for the consumption of Atlantic herring in recent years are lacking, but the co-occurrence of beluga with spring-spawning herring in the UE and fisher reports from the 1930’s suggest that Atlantic herring may be punctually important for SLE beluga in late May^79^, and possibly later in the season^28^. However, this stock (as identified in the NAFO area 4 T) has collapsed in the late 1990’s and has remained at low levels since then^80^.

Past or present abundance of capelin in the UE is unknown. Data from the LE are only available since 2008, and indicate a 20-fold decrease in catch per unit effort since then^37^, with a relative index of abundance in the northern GSL indicating below long-term average abundances since 2012^81^. Together, these result suggest an overall decrease in the availability of this lipid-rich forage species. Reasons for the change in abundance are multiple, but appear to be primarily linked to warming conditions and increased consumption by several predators, such as redfish, striped bass and pinnipeds, all of which have substantially increased in numbers over the past decades^21–23,82^**.** In this context, the future of the capelin stock remains uncertain, with few spawning events reported anecdotally in recent years in the SLE and northern GSL (https://ecapelan.ca/). A reduction in capelin availability in the SLE might explain the decrease in the consumption of pelagic prey by beluga observed in the 2020’s. This prey might be critical for beluga, hunters in the 1800’s reporting that SLE beluga gained large amounts of fat reserves in the spring while feeding on spawning capelin and/or rainbow smelt in the UE^83^. The apparent reduction in the consumption of this lipid-rich pelagic fish could have profound effects on SLE beluga energy intake, and their physiological condition, especially considering that spring feeding might be particularly crucial for pregnant females in their last term of gestation^29^. There are indications that lipid concentrations have decreased in SLE beluga blubber since the late 1990’s^84^. How the proportion of essential fatty acids and amino acids, which are both critical to pregnancy and the maintenance of an overall good health, have changed over time remains to be determined. Currently, there is no visible sign of poorer nutritional condition in pregnant females in this population^85^.

Our study also revealed that the diet of SLE beluga changed significantly over time. The increased contribution of pelagic prey from the LE to the diet of beluga in the 2020’s is consistent with the observed occupancy of the LE by SLE beluga over the past decade^27^. The limited information available on prey abundance and distribution in the St. Lawrence ecosystem suggested that these changes in diet composition, and possibly also beluga distribution, may partly reflect changes in prey availability. The striped bass^22^and redfish^86^are among the species that have increased substantially in recent years. While reasons for the apparent rebound of redfish since their collapse in the early 1990’s are not well understood, the increase noted in striped bass abundance were caused by their reintroduction in the UE and multiple stoking events from 2002 – 2017, and the expansion of the southern Gulf population following the implementation of protective measures^22,87^. The St. Lawrence river striped bass population are known to aggregate in the UE in spring, before and after spawning^88^, when a large portion of the SLE beluga population also aggregate in this area^27,89^, possibly to feed on spawning capelin, herring and rainbow smelt^28,83^. The increasing consumption of striped bass in recent years by all segments of the population is therefore consistent with their increased availability and overlapping distribution with SLE beluga.

The concentration of commercial fisheries in the Gulf of St. Lawrence and their near-absence in the SLE proper have limited long-term monitoring of stock abundance. This is particularly true for the UE where the Atlantic tomcod, an anadromous species, is the only species for which catch per unit effort are known and available since 1970^20^. Stock assessments suggest that their abundance has declined in the 1990’s and has remained relatively low since then. Similarly, the rainbow smelt, another anadromous species present in UE and the LE, underwent a reintroduction plan in 2003^90^, indicating that their abundance was also low at that time. Their small contribution to beluga diet supports a low abundance during our first time period. Their increased consumption in recent years, however, could be due to the fish stocking activities undertaken in several SLE tributaries^19,90^. While current abundance of rainbow smelt is unknown, their abundance is suspected to be at least stable or to have increased since 2003^18^. Rainbow smelt were detected in contemporary beluga digestive tracts in April, but mainly from August to October^29^. It thus cannot be excluded that beluga may be feeding more extensively on this prey in the fall, a period inadequately covered by our current sampling scheme in both time periods^28,29^.

Changes in predator abundance are know to heavily impact the trophodynamics of ecosystems by modifying top-down control^91^. However, effects of trophodynamics on foraging strategies is an understudied aspect of marine mammal ecology, and the SLE beluga is no exception. The recent increase in abundance of potential competitors of beluga for pelagic prey (e.g., several species of pinnipeds, redfish, striped bass) may have reduced prey access or availability for SLE beluga^34^. However, there may be some mechanisms acting to limit inter- and intra-specific competition as a result of population increases of competitors. Trophodynamics depend not only on resource and competitor abundance, but also on ecological opportunities. Theoretical and empirical work suggests that the rebound of one or a few species which increases species diversity may help relax inter- and intraspecific competition by enhancing ecological opportunities^6,92^. This may be a particularly crucial mechanism in a food-limited environment where potential competitors increase in abundance^93^. Striped bass and redfish may represent both competitors and prey to beluga, and whether their increase in recent years have created more ecological opportunities for SLE beluga is unknown. A comparative study estimating seasonal diet composition and niche overlap among competitors, and better data on prey availability might help understanding the trophodynamics of the SLE ecosystem, the level of intra- and inter-specific competition experienced by SLE beluga, and the mechanisms behind the observed patterns in individual dietary specialization. The differences in fine-scale habitat use between adult males and adult females and juveniles would also be highly relevant in this context as this spatial segregation may contribute to further reduce competition and enhance realized niche segregation among conspecifics and potential competitors in a given season or year, or in a specific habitat^4,94^.

Other anthropogenic factor may affect distribution differently in males and females, with a potential incidence on prey access and diet composition thereof. Vessel noise for instance can mask beluga calls, modify their acoustic behaviour and result in avoidance responses^95–97^. However, a habitat model failed to identify shipping traffic as a significant correlate of beluga habitat use in the St. Lawrence Estuary^98^. While females may be less risk-adverse than males as documented in other species^99^, there is no evidence for females avoiding the shipping lane in the SLE, which runs through both adult male habitat in the Laurentian Channel, and female and calf habitat in the UE^100^. It is worth nothing, however, that in 2020 when COVID-19 reduced vessel traffic in many areas including the SLE, diet composition, and the importance of pelagic prey from the LE in the diet somewhat differed compared to other years within the period (2015 – 2020). Whether the observed variation was related or not to the reduction in traffic and greater access to valuable habitats is unknown.

In conclusion, our study provided new insights into how SLE beluga have changed their feeding behavior to cope with trophodynamic changes in the SLE over the past three decades. While the functional group of pelagic prey remains the primary resource for most individuals, at least in spring and early summer, our results indicate a decrease in the contribution of pelagic prey to the diet, and a larger heterogeneity in diet composition within specific age or sex classes, suggesting a diet diversification both at the individual and population levels. These findings suggest that specific underlying processes have acted, or are still acting, on the niche structure of this endangered population, with likely effects on their energetic budgets. Whether these patterns emerged from an environment-driven reduction in prey biomass or quality, or from an increase in intra- and/or inter-specific competition is unknown. They, however, underscore the importance of considering not only the population, but also individuals when studying the foraging ecology of endangered species where every individual count.

## Supplementary Information

Supplementary Information 1.

Supplementary Information 2.

Supplementary Information 3.

Supplementary Information 4.

Supplementary Information 5.

## Data Availability

All datasets (i.e., isotopic data on individual beluga and potential prey) that support the findings of this study as well as supplementary tables are available online as supplementary information files or from the corresponding author upon reasonable request.
