# Supplementary material for: Changing ecosystems promote generalism and enhanced heterogeneity in diet composition in the endangered St. Lawrence Estuary beluga
Source: Sci Rep. 2025 Feb 20;15:6239. doi: 10.1038/s41598-025-91083-z (PMC11842592; doi:10.1038/s41598-025-91083-z)
Supplement: Supplementary file 2 — Supplementary Information 2. [file 41598_2025_91083_MOESM2_ESM.docx]

Response to reviewer Comments:

We would like to express our sincere gratitude to both reviewers for their time and valuable comments. We have incorporated their feedback directly into the manuscript using track changes and provided responses to each comment. To facilitate the review process, our responses are highlighted in color. We are also providing a "clean" version of the Word document.

Reviewer 1

Dear Authors,

I enjoyed reading your manuscript. It was written well, and I found the research informative and interesting. Certainly, worthy of publication. Not all of the following comments require a change to manuscript so please keep this in mind as you read them.

Line 38 - On a similar note, are there documented increases in species who feed similar to beluga in these areas over these same time periods? This is mentioned to some extent but could be elaborated on.

No other cetacean species in the St. Lawrence have as diverse a diet as the beluga. However, some of our research has shown that other cetacean species, particularly fin whales, which primarily feed on pelagic fish (e.g., capelin) and krill, diversified their diet between 1998–2006 (Cabrol et al. 2021). This likely reduced intra- and/or interspecific competition. We discuss this aspect in more detail in the discussion (lines 334-337), as the first paragraph of the introduction is rather general and focused on general ecological theory.

Regarding the development of marginal behaviors, except for the grey seal, there are currently no other study showing the presence of marginal behaviors in marine mammals in the St. Lawrence.

Reference :

Cabrol, J. et al. Individual and population dietary specialization decline in fin whales during a period of ecosystem shift. Sci. Rep. 11, 1–14 (2021).

Line 39 - Is it possible to examine changes in water traffic between the two time periods? Could an increase in boating traffic be a possible cause of interspecific differences in feeding? Males in other species are shown as being less risk-averse when compared to females and young, perhaps this may be a possible cause here? Could watercraft traffic increases mimic an intraspecific "structuring effect", by driving certain risk adverse individuals to alternative feeding areas?

We have added a paragraph to discuss this interesting point (lines 489 – 500). However, there is currently no evidence of such an effect in the SLE, based on habitat model outputs where vessel traffic as a driver of beluga habitat use in the St. Lawrence Estuary was tested along with other factors (Mosnier et al. 2016). Shipping lanes cross the habitat of both adult males and females and calves and so, expose both sexes to the same amount of shipping with no indication of female avoiding these areas.

Reference :

Mosnier, A. et al. Definition and characterisation of the St. Lawrence Estuary beluga (*Delphinapterus leucas*) habitat following an ecosystemic approach. *DFO Can. Sci. Advis. Sec. Res. Doc*. **2016/052,** vi + 93 p. (2016).

Line 139 - "... δ15N determination were also done using lipid-extracted samples at the time. " Is unclear, consider re-writing.

We modified the sentence as suggested.

Line 291 - Is the change in 2020 due to decreases in activity during the covid-19 pandemic?

Our time series was from 2015 to 2020, with COVID potentially affecting our last year of data. Variability in pelagic prey consumption was lower among individuals that year (figure 3), and diet composition did come out as somewhat different from the other years within that period. However, the mean diet or specialization index in the 2020’s were still strikingly different from the 2000’s suggesting no major effects of year 2020 on our conclusions. We added text to the Discussion to highlight this point about potential covid-related effects (Starting line 496).

Line 397 - Is the possible change in habitat referring to the habitat for the fish or for the beluga or both. This is unclear to me.

In line 396, “habitat use” referred to the pelagic fish. We changed the sentence accordingly.

Reviewer 2

This study combines stable isotope analysis of muscle samples from stranded belugas in the St. Lawrence Estuary with isotope analysis of prey in a mixing model framework to provide estimates of diet composition and identify factors influencing diet variation at two time periods. Overall, the study was fairly well described and the authors were able to achieve a good sample size for belugas, as well as good spatial temporal coverage of prey samples, which helps bolster their conclusions. There were a few areas that could use some clarification and refinement, particularly in parts of the methods and in in the discussion. I also think some additional clarification of the specialization index is warranted, since its original description was as an indicator of population-level specialization and not individual specialization. I have provided some line-specific comments below for consideration during revision. I also had some minor editing suggestions in a word document, however, it appears there is not a place to upload attachments so I have tried to detail some of those below as well.

Lines 60 - 61: Suggest moving this up in the paragraph to mention the environmental shifts first, since it is likely these are the underlying factors driving the upper trophic level changes.

Moved up (lines 51-55).

Lines 69 - 71: Suggest reversing order of this sentence - i.e. these changes have altered the niche breadth and dietary overlap of potential prey species but it is unknown how they have affected the trophodynamics and trophic niche of marine mammals.

Modified as suggested (lines 62 to 63).

Line 91: Suggest replacing "little to no contents" with "largely empty stomachs"

Changed (line 84)

Lines 106 - 107: Suggest clarifying here what the environmental conditions were during the 2000s period. Is it just that it was colder?

We added information regarding trophodynamic between both periods (lines 99 to 101)

Line 150: This should read "the mantle"

Changed

Line 175: Can you specify whether you used data specific to their region to correct values or one of the preset regions in this package?

We used the preset for the north Atlantic region as indicated in the Suess Package. Sentence has been modified as accordingly (line 165)

Lines 196 - 197: Consider including information on what packages you used to run analyses. Also, is this statement true for all the analyses or just for this section?

Sure. We added information on Vegan package (line 169)

Line 202: Is the R package MixSiar?

Indeed. We modified the sentence to explicitly mention the R package (line 183).

Line 204: It is unclear what the individual beluga here refers to in the context of sampling year.

I assumed that this comment refers to the sentence on “…individual beluga: n = 96), with random effects only (individual beluga)…..” in line 189. Here, individual beluga does not refer to the sampling years, but to the factor used. We used individual beluga as a random factor. Depending on the model being tested, they could be used alone as a random factor, as in the model “individual,” or nested within the year (Table 1). However, to avoid confusion, we modified this sentence.

Lines 219 - 220: Would this sentence be more appropriate following mention of how the models were parameterized?

We agreed. The sentence on the goodness-of-fit has been moved down after the section on model parametrization (starting at line 203).

Lines 219 - 237: I think it would be helpful to preface the information in these two paragraphs with a brief general statement (~ 1- 2 sentences) about inputs to the mixing model. This would just help provide some context for the information presented here.

We agreed. However, we decided to add subtitles rather than a general sentence. We thought it would be easier for readers.

Lines 240 - 241: Suggest including the citation that actually describes this metric, which is Newsome et al 2012, as cited in reference 65.

Added (line 220).

Also, as stated in Newsome et al 2012, this metric describes the specialization index for a population, not an individual, which would seem to indicate this is not the correct metric to represent individual diet specialization. If the authors are applying it in a new way, then some additional description is needed, although much of the results/conclusions seem like they are more in line with this being used to represent a population level specialization index. That is, the diet of belugas was dominated by pelagic prey in the first time period but more mixed among prey groups in the second, suggesting a population-level switch from specialists to more generalists. If the latter is the case, some rewording is necessary to convey that the authors are focused on specialization at the population level and/or at the level of the age classes.

We agree this paragraph could have been misleading and we modified it accordingly (line 220-226). As suggested by the reviewer with used the Ɛ to compute the population index level using the estimated diet of individual belugas.

Line 281: The inclusion of (3 levels) here is confusing. These have already been defined previously so I don't think it is necessary to include it here.

Deleted

Lines 283 - 285: I found this a little bit confusing. The first model also included sex and age class (so what is different? no random effect of individual?) Also what is the random effect referenced here?

We clarified this paragraph and now indicate what the random effect refers to (line 260 to 263)

Lines 385: Can you clarify what you mean by "these areas largely serve foraging functions". Perhaps just simplify by just stating that these are indicative of foraging areas?

We modified as suggested (line 342).

Lines 386: Can you clarify what you mean by "characteristics"? Environmental, prey assemblages, etc?

We now specify why these areas differed (line 341 to 343).

Lines 396 - 422: I would suggest combining this information into a more succinct single paragraph, as it seems unnecessary to have two paragraphs describing why individuals are likely to vary in their diets. For me, this was a very minor part of the manuscript so doesn't need extensive description.

We agree. As suggested with screened the paragraph and changed focus on the individual (starting line 337).

Line 399 - 400: I found this statement here, that these habitats likely support different prey assemblages to be a bit confusing, since it was already stated in Line 397 that they have different community structure.

The whole paragraph has been modified according to the previous comment.

Lines 400 - 402: Sulfur stable isotopes might also be useful in partitioning pelagic vs estuarine. Perhaps something to mention in terms of future research directions.

We added sulfur as potential marker (line 356)

Lines 439 - 441: Consider also including the information in the methods, so the reader has an idea of what this is ahead of time

Since the line numbers in the comment differ from those in the annotated (or original) document, we were unable to determine which information the reviewer wished to see repeated here.

Discussion: The discussion jumps around quite a bit from topic to topic and I think some reorganization would help with the flow. There also seemed to be a lot of disproportionate focus on individuals, which seemed like a very minor part of the paper. In contrast, while the authors did discuss changes in diet between the two time periods, mention of the fact that individual year was important in more recent models seems warranted, as this provides some important indicators in regards to the consistency in available prey resources (or lack thereof) for SLE belugas.

We changed some of the sections of the discussion to help with the flow. In addition, we decreased the focus on the individual as suggested.

Table 1: I found some of the formatting to be confusing here, such as the use of quotations around sex and age class and the inclusion of individual in parentheses. Also, is the "x" intended to represent an interaction between year and sex/age class? Also, only 8 models are listed but in the text it said 10?

Thank you for pointing this out. We tested eight models and have corrected this throughout the manuscript. Regarding notation, the “x” refers to the interaction, and we quote sex/age classes to avoid any misunderstanding, as some might think we have two distinct factors (Age * class) instead of one factor.

Please let us know if there is a preferred or more formal way you would like to see the model formulation, and we will gladly modify the paper.

Figure 3a. As currently organized, with period in a separate subplot, it hard to visualize what appears to be the main purpose of this figure, to highlight the differences in diet between periods. I would consider making this one larger plot where the two time periods are shown next to each other for each prey group. That or potentially adding a third panel that is the difference between the two to really make it clear.

There was a number of considerations in the balance when we decided on the format of this specific figure. We refrained from going in the direction proposed because we felt that the comparison between the two periods was striking enough to be picked up with the current figure. However, the effect size for variability among age and sex classes or among sampling years within a period would be much more difficult to see from a figure as proposed by the reviewer. For all these reasons, we would prefer to maintain the figure as currently presented.
